# Differential expression of miR-4520a is associated with gain of function mutations in Familial Mediterranean Fever (FMF)

**DOI:** 10.1186/1546-0096-13-S1-O10

**Published:** 2015-09-28

**Authors:** H Latsoudis, MF Mashreghi, J Gruen, H-D Chang, B Stuhlmueller, A Repa, I Gergiannaki, E Kabouraki, P Papakosta, T Haeupl, A Radbruch, P Sidiropoulos, D Kardassis, D Boumpas, G Goulielmos

**Affiliations:** 1 University of Crete, Medical School, Internal Medicine, Heraklion, Greece; 2 German Rheumatism Research Center (DRFZ), Berlin, Germany; 3 German Rheumatism Research Center (DRFZ), Bioinformatics, Berlin, Germany; 4 Charité University Hospital, Rheumatology and Clinical Immunology, Berlin, Germany; 5 University Hospital of Heraklion, Clinic of Rheumatology, Heraklion, Greece; 6 University of Crete, Medical School, Biochemistry, Heraklion, Greece; 7 Institute of Molecular Biology and Biotechnology, Foundation for research and Technology of Hellas, Heraklion, Greece; 8 University of Athens, Medical School, Athens, Greece

## Introduction

MicroRNA signature of THP1 cells revealed a 5.9-fold decreased expression of miR-4520a following siRNA-mediated knockdown of *MEFV* gene that encodes pyrin [1].

## Objectives

We herein sought to validate the expression levels of miR-4520a in monocytes isolated from peripheral blood mononuclear cells (PBMCs) of FMF patients.

## Methods

Dual luciferase assay was used to validate a predicted miR-4520a recognition element in the 3'UTR region of the Rheb gene. The expression levels of pyrin, miR-4520a and its putative target Rheb were validated in monocytes from FMF patients (n=9) and compared with healthy controls (n=8). Patients were off colchicine for two days (attack-free period) and monocytes were isolated from PBMCs. Total RNA together with the respective miRNA-enriched fractions were isolated from monocytes and used for mRNA and miR-4520a quantitation by real-time PCR using the 2-ΔΔCt method after normalizing to 18S RNA and RNU6B genes, respectively. Protein levels of pyrin and Rheb were detected by western blotting.

## Results

The relative expression levels of miR-4520a were variable among FMF patients and not significantly different between patients and controls. However, when patients that did not harbor any mutations in *MEFV* were excluded from the analyses, the expression of miR-4520a was statistically different between FMF patients and controls (p<0.05), indicating an association between miR-4520a expression and mutations in the *MEFV* gene. Moreover, stratification of patients group by genotype revealed an intriguing difference in miR-4520a relative expression, with carriers of M694V variant (combined group of homozygotes, heterozygotes and compound heterozygotes) showing the highest increase (p<0.05). Subsequent comparison between the M694V group and healthy controls showed a significant increase in miR-4520a expression levels that remained significant even after bonferroni correction (p<0.01). Interestingly, one of the homozygote M694V patients with the highest fold change in miR-4520a expression (FC=7.8) experienced an FMF-attack while on study, with a concomitant decrease in miR-4520a relative expression (FC=0.45). Bio-informatic analyses showed that miR-4520a is predicted to target genes implicated in autophagy through regulation of Rheb/mTOR signaling. Expression levels of Rheb were confirmed by luciferase reporter gene assays providing further evidence that Rheb is a direct target of miR-4520a (p<0.01). Validation of pyrin and Rheb protein expression levels in monocytes from FMF patients is in progress.

## Conclusion

Our findings provide initial evidence that Rheb is a valid target of miR-4520a and suggest that a dysfunctional pyrin due to gain of function mutations with a dosage effect [2], especially of M694V variant, may be associated with an increase in miR-4520a expression levels, thus contributing to deregulated mTOR signaling and subsequently IL-1β release [3].
